# Prevention of Pediatric Respiratory Syncytial Virus Lower Respiratory Tract Illness: Perspectives for the Next Decade

**DOI:** 10.3389/fimmu.2019.01006

**Published:** 2019-05-07

**Authors:** Sofia S. Aranda, Fernando P. Polack

**Affiliations:** Fundacion Infant, Buenos Aires, Argentina

**Keywords:** RSV, RSV vaccines, monoclonal antibodies, maternal immunization, live attenuated vaccines

## Abstract

The landscape of infant bronchiolitis and viral pneumonia may be altered by preventive interventions against respiratory syncytial virus under evaluation today. Pediatric wards in 2018 in developing countries may differ from those attended by future generation pediatricians who may not witness the packed emergency rooms, lack of available beds, or emergency situations that all physicians caring for children with RSV experience every year. In this review, we describe and discuss different prevention strategies under evaluation to protect pediatric patients. Then, we outline a number of potential challenges, benefits, and concerns that may result from successful interventions after licensure.

In the next decade, we may witness a change in the burden of office and emergency room visits, and hospitalizations in infant wards during the winter. Swamped emergency rooms and pediatricians and nurses overwhelmed with work, caring for an endless number of young infants in respiratory distress may start to become a scene of the past. Vaccines, monoclonal antibodies of extended half-life and antivirals against respiratory syncytial virus (RSV) are being evaluated in clinical trials (1). It is reasonable to expect that several of these candidates will be partly or completely successful in the next 10 years and become part of the preventive and therapeutic tools for pediatric public health.

RSV is the main cause of hospitalization in infants and young children worldwide. Millions of children are hospitalized every year and the vast majority of them live in the developing world. While infants born prematurely, those with congenital heart disease and other specific subgroups are at increased risk for hospitalization, the majority of severe cases affect previously healthy infants and children. The only available intervention licensed to prevent severe disease today is the administration of a neutralizing anti-RSV humanized monoclonal antibody, palivizumab®. Palivizumab® is recommended for high risk populations in high and middle-income countries (2). But its cost is prohibitive for low income nations, where most of the fatal cases of RSV disease occur.

Evidently, a safe and effective, affordable preventive strategy against RSV is necessary. One of these strategies under evaluation is to immunize pregnant women against the virus to transfer high titers of protective antibody to infants before birth (1). Alternatively, long-lived monoclonal antibodies against neutralizing epitopes in the RSV F protein could be administered to newborns and young infants to prevent disease in the first months of life (1). Other attractive approaches under study include immunization of infants with recombinant live attenuated RSV vaccines or using a variety of live vectors carrying genes encoding RSV proteins (1). Even though natural infection with RSV does not induce lifelong protective immunity, antibodies against the RSV F glycoprotein can prevent severe disease in humans. RSV F is a target for neutralizing antibodies, as evidenced by the effectiveness of palivizumab for nearly two decades. Hence, F alone or in combination with other viral proteins is the preferred antigen in RSV candidate vaccines (3).

## Eliciting Protective Immunity Against RSV LRTI

Humans are the only natural host for RSV. The virus is spread from person to person via respiratory droplets, and spreads into the respiratory tract, where it preferentially targets apical ciliated epithelial cells (4). The incubation period for RSV from time of infection to onset of illness is between 3 and 5 days (5). Natural immunity includes innate responses by polymorphonuclear (PMN) and mononuclear cells, activation of numerous pattern recognition receptors (e.g., TLR3, TLR2/6, TLR7/8, NOD-like, and RIG-I-like receptors), and type I and III interferon responses (6–9). These responses are important, as PMNs and macrophages have been postulated to enhance and prevent severe disease, and PRRs have been reported to modulate numerous responses during RSV infection (5, 10). Moreover, although often underappreciated because their levels peak before RSV symptoms become evident to pediatricians, IFNs are increasingly recognized as relevant actors in RSV immunity (9, 11). In phase I trials with intranasal live attenuated RSV vaccines in seronegative infants, a small but significant number of subjects is not infected despite direct inoculation, suggesting that innate immune responses may be an important barrier against infection.

Adaptive immunity is critical for protection against RSV disease. The humanized monoclonal antibody palivizumab® and polyclonal sera enriched for antibodies against the RSV fusion (F) protein, Respigam®, demonstrate that antibodies against RSV F can prevent severe RSV LRTI. RSV infection elicits polyclonal, high avidity, neutralizing antibody responses against RSV F (12), that cross-react between RSV subgroups A and B. The F protein is highly conserved between RSV subgroups, with amino acid sequence identities of >90% (5, 13). This protein mediates entry into host cells by converting from a metastable trimeric pre-fusion conformation (pre-F) to a highly stable post-fusion conformation (post-F) (14). There are two pre-F-specific antigenic sites Ø and V, target of the most potent neutralizing antibodies, and two sites that are present on both conformations II and IV (12, 14). Although present on both pre-F and post-F, antibodies against site III bind tighter to pre-F (14). Instead, site I antibodies bind tighter to post-F (14). A newly recognized antigenic site in F, designated antigenic site VIII, occupies an intermediate position between the previously defined sites II and Ø (15).

The RSV attachment (G) protein also elicits polyclonal neutralizing responses. RSV G is more variable than RSV F, with ~50% sequence homology between subgroups A and B (5). Both RSV subgroups co-circulate during yearly epidemics, although one typically predominates every season (16–18). While IgA responses are probably important in protecting the respiratory tract, their exact role in RSV immunity is only now becoming clearer and requires further study (11).

T cell responses are also important for protection against RSV. CD4^+^ T lymphocytes contribute to T-cell dependent antibody responses and indirectly to viral clearance (as evidenced by persistent infections in HIV-infected individuals) and CD8^+^ T lymphocytes clear the virus from infected cells (5, 19).

The aforementioned innate and adaptive immune responses are mimicked to different degrees by live attenuated vaccines against RSV, discussed below. But in specific situations, immune responses against RSV -particularly those elicited by non-replicating vaccines in naive individuals- can also potentiate disease severity.

The first time an enhanced form of RSV disease (ERD) was observed in children during a vaccine trial, its manifestations were not recognized by investigators. Between 1962 and 1963, a formalin-inactivated vaccine against RSV (FIRSV) was evaluated in 54 children in the United States. Unfortunately, none of them received placebo. Twenty-one of fifty-four (39%) vaccine recipients were infected with RSV, and 10 (18%) experienced severe disease. But only 5 years had passed since the isolation and initial characterization of the chimpanzee coryza agent (now RSV), and its burden of illness was still unclear (20). As a consequence, researchers attributed the observed severity to an unusually bad season (20).

**Figure 1:**
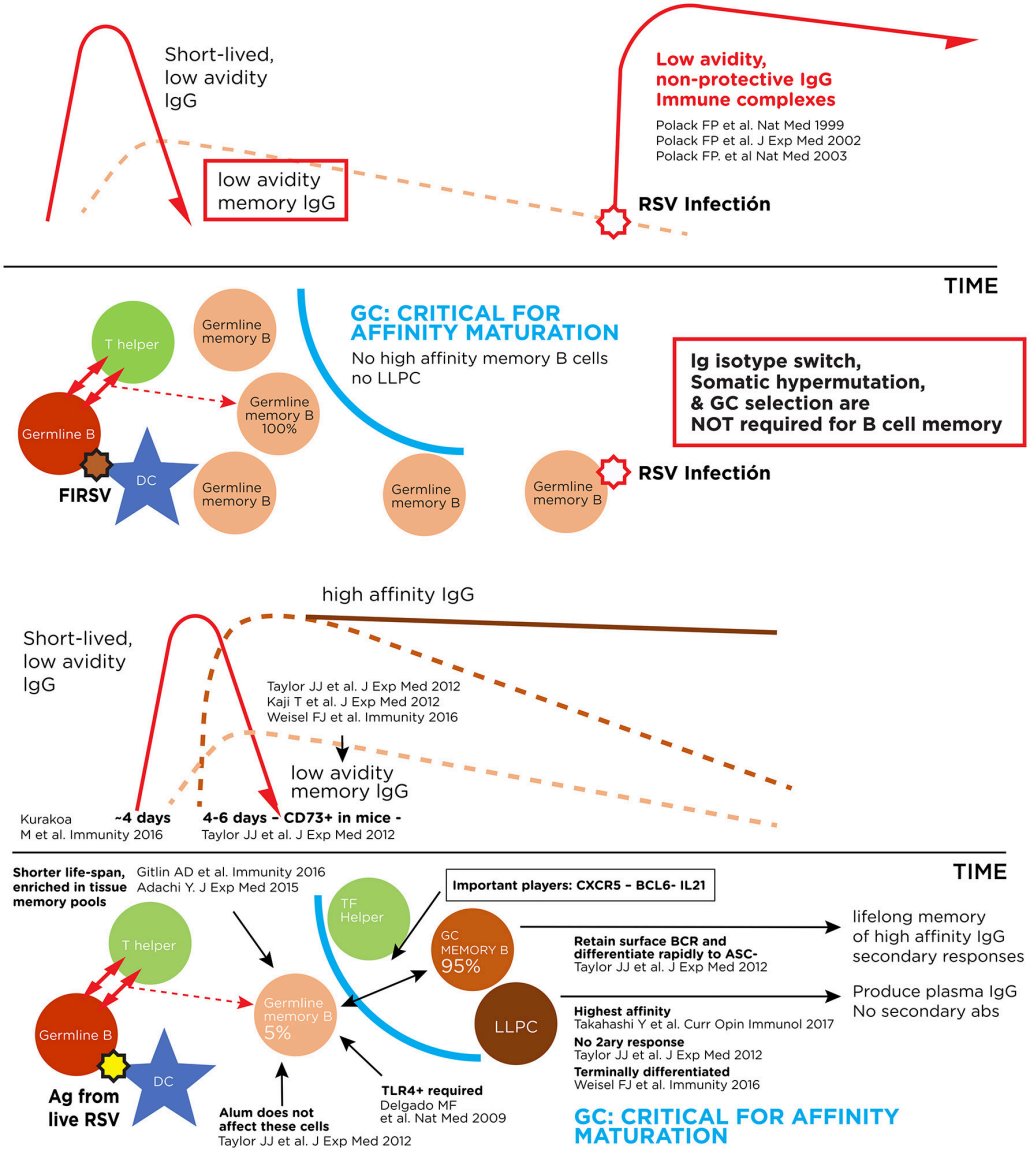
Pathogenic and protective antibody responses against RSV. Upper panel: Immune complex formation with antibodies of low affinity for the virus. Lower panel: Antibody response to live RSV infection with production of high affinity long-lived memory B cells and long-lived plasma cells (LLPC).

In 1966, a similar formalin-inactivated vaccine against RSV was administered to infants and toddlers in four trials in the United States. Immunized subjects had measurable non-neutralizing, low avidity anti-F IgG responses, no RSV-specific cytotoxic T lymphocytes and a CD4^+^T lymphocyte response primed to respond to wild type infection with an exuberant production of Th2 cytokines (10, 21–23). Indeed, upon infection with wild type RSV during the winter of 1966-1967, non-protective, low avidity IgG coupled with the virus to activate the complement cascade and in synergy with a strong Th2 polarization of the immune response led to increased hospitalization rates and two deaths in toddlers due to this enhanced form of RSV disease presenting with wheezing and bronchopneumonia (20, 24–26). Consequently, generation of an IgG response dominated by low avidity, non-neutralizing antibodies against RSV F (Figure 1), and priming for a Th2 bias upon RSV infection are considered undesirable features for RSV vaccine candidates.

## Immunizing Mothers to Protect Young Infants

Maternal immunization, a strategy well-accepted to prevent infant influenza and pertussis, aims to provide passive immunity to infants by transfer of maternal antibodies across the placenta. During the third trimester of pregnancy, IgG antibodies are actively transferred via the FcRn receptor in the placenta from the maternal to the fetal circulation. The first candidate in clinical trials to complete phase III evaluation in eleven countries enrolling approximately 4,600 women of childbearing age was the Novavax prefusogenic RSV F nanoparticle adjuvanted with alum (NCT02624947) (27). The Novavax vaccine elicited responses against epitopes displayed by both pre-fusion and post-fusion conformations of the F protein (28). The trial aimed to reduce the rate of medically significant RSV LRTI in infants through 90 days of life (27), but failed its primary endpoint despite an overall efficacy of 39.4% (95% CI, 5.3, 61.2) (29). Interestingly, the vaccine protected against RSV hospitalizations (40%) and severe disease (44%) worldwide (29), but its efficacy was radically different in the U.S. compared to developing countries: results in South Africa were considerably better than in the American population at 57% (95% CI, 32.7, 72.5) vs.−32.7% (95% CI,−238.9, 48.1). Unfortunately, the study lacked sufficient power to confidently ascertain whether these differences simply represented variations within the confidence interval.

Another vaccine designed for maternal immunization is a recombinant RSV protein F vaccine, engineered to preferentially maintain prefusion conformation by GSK. The vaccine progressed through early phase clinical trials in healthy adults and non-pregnant women (NCT02753413) (30–32). In addition, a subunit vaccine encoding a stabilized prefusion molecule developed by Pfizer also targets pregnant women (and the elderly) and is entering phase 2 trials (NCT03529773) (33–35). A fourth attractive investigational candidate developed by NIAID and entering clinical evaluation is RSV F DS-Cav1 (NCT03049488) (36), a formulation of engineered soluble site Ø–stabilized RSV F trimers (site Ø is only present on pre-fusion F) adjuvanted with alum (37, 38).

## Long-Lived Monoclonal Antibodies

The concept of administering an optimized mAb with extended half-life and strong neutralizing activity to protect infants after birth is appealing. MEDI8897 from Medimmune is a highly potent, extended half-life antibody that completed phase 2 trials and is expected to enter phase 3 in 2019 (NCT02878330) (39). Recently, MEDI8897 was granted breakthrough therapy designation by the FDA. MEDI8897 is a recombinant human RSV monoclonal antibody with a modified Fc region. The antibody has been optimized from antibody D25, which targets site Ø in the pre-F conformation of the RSV F protein. MEDI8897, with a mean half-life 85 to 117 days, is intended to prevent RSV disease in all infants for the duration of the RSV season with a single dose (40). A triple-amino-acid (M252Y/S254T/T256E [YTE]) substitution within its Fc region extends its half-life by increasing binding to the major histocompatibility complex class I-related neonatal Fc receptor (FcRn) at acidic conditions and preventing degradation (40–42). A second long-lived monoclonal antibody candidate in clinical development is MK-1654 from Merck (NCT03524118) (43), undergoing early phase evaluations and targeting site IV in RSV F.

Despite the intuitive attraction of using a targeted mAb to neutralize RSV and prevent severe illness, this virus has proven once and again to be an annoying creature. A recent phase 3 study evaluating suptavumab (REGN2222), an antibody developed by Regeneron against site V in RSV F, did not meet its primary efficacy endpoint in healthy preterm infants (NCT02325791) (44). REGN2222 had a non-significant protective trend against medically attended infections in preterm infants up to day 150 of life caused by RSV subgroup A. But due to an unexpected mutation in site V of RSV F in circulating RSV B viruses, failed to prevent disease against this subgroup (44).

## Live-Attenuated Vaccines Against RSV

Several live-attenuated RSV vaccines candidates to protect infants and young children are in clinical development (1). One of their strengths as infant vaccines is that decades of studies in infants have shown that these vaccines do not appear to prime for enhanced RSV disease in RSV-naïve infants (45). In addition, these vaccines are administrated intranasally, needle-free and, given that they replicate in the upper respiratory tract, can generate an immune response even in the presence of passively acquired maternal antibodies (46). Their greatest challenge is to attain the adequate balance between attenuation and immunogenicity. A clever deletion of the coding sequence for the RSV M2-2 protein in one of these candidates attenuates viral replication while upregulating gene transcription and antigen expression (NCT02237209, NCT02040831) (47, 48). The NS2 non-structural protein was deleted in a second candidate(NCT03422237, NCT03099291) (49, 50) reducing viral suppression of type interferon responses in the host (51). Both candidates were developed by the Laboratory of Infectious Diseases at NIAID and were safe in early studies in infants and young children and may soon progress to larger trials for evaluation. While no head-to-head comparison was described, the similarities in trial design suggest that the M2-2 vaccine has more restricted virus shedding but was more immunogenic than the live vaccine with the NS2 deletion (52).

## Vectors Encoding RSV Genes

Vectored-based vaccines for children are now in clinical trials, using adenoviruses. Adenoviruses are highly immunogenic and induce both innate and adaptive immune responses (53). Moreover, adenovirus-based vaccines have been and are currently being investigated as vectors targeting viral, bacterial, and protozoan pathogens (53). The candidate Ad26.RSV.preF uses a human adenovirus 26 expressing pre-F RSV protein. Ad26.RSV.preF is now in phase 2 trials in adults and 12-24-month-old RSV seropositive toddlers (NCT03303625) (54). A second adenovirus-based candidate in phase 2 trials in seropositive children is ChAd155-RSV (NCT02927873) (55), using a replication-incompetent chimpanzee adenovirus 155. This vector encodes the F, N, and M2-1 RSV proteins (51, 55).

Finally, a chimeric candidate in clinical development is the rBCG-N-hRSV vaccine (NCT03213405) (56). A recombinant BCG expressing RSV N protein is targeted for use in newborns. BCG induces Th1 immunity, skewing responses away from undesirable Th2 priming (57). Vaccination with rBCG-N-hRSV is expected to elicit cellular immunity in addition to a humoral response against the virus (58).

## The Future

Whether one or many of these vaccines and/or mAbs prove effective, several questions, concerns, and speculations remain to be answered through the trials and subsequent studies post-licensure. Some of these issues are discussed below.

## Concerns of Enhanced RSV Disease

The mechanism of illness of ERD has not been completely elucidated, in part because every significant scientific advancement in structural virology and immunology continues to uncover new angles of a complex problem. For several years, two immune correlates described above have been accepted as indicators of candidate RSV vaccines that may prime for enhancement: the presence of low avidity, non-protective antibodies after immunization (Figure 1) (10, 21, 59), and a Th2 polarization of the immune response in the respiratory tract after RSV infection (23, 60). Now, novel observations in the field suggest a potential relationship between RSV F conformations in the vaccine and disease enhancement (14, 61); experience with other immunogens question whether route of vaccination may be an important determinant of RSV vaccine responses (62); improved understanding of B cell memory, class switching and affinity maturation may allow a clearer identification of primed B cell memory populations associated with undesirable outcomes (63–65); and detection of subpopulations with genetic mutations in molecules essential to B and T cell maturation interrogate whether enhancement could ever be possible in seropositive adults (66–68).

Importantly, given that memory B and T helper cells play a critical role in ERD pathogenesis, concerns for this problem are lower in several leading approaches to RSV prophylaxis that rely on passive acquisition of antibody: immunization of pregnant women to protect infants through transplacental transfer of antibody and administration of virus-specific monoclonal antibodies (mAb) of extended half-life (69, 70). Moreover, a series of maternal immunization studies and years of clinical experience with mAb in vulnerable infants never identified a case of ERD (2, 71, 72). Finally, infant intranasal immunization with live attenuated RSV vaccines (LAV) mimics natural infection, and after extensive testing in early phase trials were never found to associate with ERD in seronegative subjects (45).

Perhaps the greatest challenge will come from novel platforms that defy our traditional vaccine testing paradigms and may also alter our criteria for discriminating ERD in the future. For example, vaccine replication may not be necessary to prevent ERD priming in PAMP-adjuvanted vaccines (10, 73, 74), or a stabilized pre-fusion RSV F may elicit protective antibodies of high affinity.

## Endotypes in RSV LRTI

The diversity in clinical presentations, combination of signs and symptoms, and variety in long-term consequences strongly suggest that RSV LRTI is not a single disease. In fact, it is probably a collective noun used to describe a set of clinical signs, which may obey different pathophysiological mechanisms. While subtypes of LRTI sharing similar observable characteristics are often designed as phenotypes, endotypes identify discrete subtypes based on specific mechanisms of illness. For example, middle-class urban and suburban infants with loss-of-function single nucleotide polymorphisms in Asp299Gly and/or Thr399Ile (TLR4^+/−^) are severely ill when infected with RSV due to an exaggerated Th2 responses in the respiratory tract. Moreover, these infants are not protected by the administration of RSV-specific mAb when premature (75). Children in Navajo and Apache reservations are also particularly susceptible to RSV (76). And a high-affinity mAb against RSV failed to prevent long-term recurrent wheezing in them, despite reducing the rate of severe acute RSV disease (77). Alaskan native children are equally susceptible to acute infection with the virus. Therefore, efficacy and subsequent effectiveness of preventive strategies against RSV may differ in certain RSV LRTI endotypes, and be affected by environmental exposures, population genotype, and/or circulating viruses.

## Viral Replacement or Extended Protection

An interesting dilemma is whether a successful vaccine or mAb against RSV will lead to replacement of the virus by a different pathogen as a cause of pediatric illness or vaccination will impact other pulmonary ailments currently not known to be triggered by RSV.

In principle, vaccines and mAbs are not expected to prevent RSV infection in the upper respiratory tract, and therefore other agents would still compete for the niche with RSV in vaccinated subjects (78). However, recent data suggest that, upon prevention of RSV LRTI with palivizumab, other respiratory viruses may indeed replace a portion of the LRTI burden (78). A recent study in 429 premature infants, described a reduction in RSV LRTI, but reported no differences in the absolute number of respiratory episodes (78). These observations paralleled an increased rate of rhinovirus infections in recipients of palivizumab (79). A possible explanation may be that infants intermittently experience “*windows of susceptibility*” that allow pathogens to cause disease. RSV may outcompete other viruses for the nasopharyngeal niche in early life, but following immunization (or administration of mAb), infectivity of RSV may be “weakened”. Then, other viruses may outcompete this “weakened” version, becoming more frequent agents of LRTI. Therefore, it is conceivable that some of the burden caused today by RSV will be elicited in the future by other viral pathogens.

Interestingly, the maternal Novavax RSV prefusogenic vaccine evaluated worldwide conferred significant protection against all-cause LRTI (25.3%) and all-cause LRTI with significant hypoxemia (<92% O_2_ sat; 39.1%). Therefore, while it is conceivable that RSV may be replaced in certain situations, recent data suggest that significant, partly unexpected benefits may follow transplacental acquisition of maternal antibody in infants in developing nations.

## Reducing Burden of Recurrent Wheezing and Asthma

RSV LRTI in infants and young children associates with 25–80% greater subsequent rates of recurrent wheezing and asthma when compared to children not experiencing severe RSV LRTI (80, 81). And while some of these RSV lung ailments affect infants genetically predisposed to develop asthma at an older age (82), severe RSV LRTI may also contribute to the inception of recurrent wheezing and asthma in children (80, 82). In fact, enough evidence exists today to prompt long term follow up of vaccinated subjects in clinical trials to ascertain a potential role for RSV vaccines in decreasing the burden of recurrent wheezing (77, 78, 83, 84).

A variety of studies in premature infants examined the efficacy or effectiveness of palivizumab® in preventing long term wheezing and asthma at ages 1 and 6 years (78, 83–86). Most of them described a protective role for RSV prevention against subsequent episodes of wheezing in infancy (78, 83, 84). The long-term effect elicited by RSV appears to be specific, and not triggered by infections with other viruses (78). However, a similar study with a virus-specific mAb in term, healthy Native Americans in Arizona prevented severe acute RSV LRTI but had no effect on rates of medically attended wheezing in children aged 1-3 years highlighting the potential importance of “endotypes” in future results (77).

At age 6 years in the randomized clinical trial in premature infants in The Netherlands, treatment significantly reduced parent-reported current asthma. The observation was based on different rates in infrequent wheezing (1-3 episodes/ year) (85), while physician diagnosed asthma and lung function were not different in drug and placebo recipients (85). In Japan, a second study revealed that palivizumab® prophylaxis administered to preterm infants did not suppress atopic asthma but lowered the incidence of recurrent wheezing (86).

Ongoing RCTs constitute a unique opportunity—perhaps the only ever- to settle these questions and determine the role of RSV in asthma inception. In recent years, a group of investigators reported guidelines for the evaluation of recurrent wheezing and asthma in upcoming studies (87). Yet, we must remain mindful of the fact that asthma is also a set of heterogeneous diseases sharing common symptoms. Therefore, it is highly likely that preventing severe RSV LRTI may affect one or few of these asthma endotypes but not others. And only a more sophisticated discrimination of the diseases under the “RSV LRTI and asthma umbrellas” will allow a definitive understanding of the mechanistic associations between both syndromes.

## Decreasing Infant Mortality

In 2015, more than three million children were hospitalized with RSV LRTI and up to 118,000 died at hospitals and in the community. Ninety-nine percent of deaths occurred in developing countries (88). Deaths attributable to RSV in industrialized countries are not frequent, affecting children with congenital heart disease, chronic lung illness, neuromuscular disorders or genetic syndromes (89). In contrast, healthy term infants from socially vulnerable environments in the developing world die at the hospital with bacterial sepsis and/or affected by clinically significant pneumothoraxes (90). Importantly, approximately 50% of deaths due to RSV in the developing world are known to occur at home. These deaths affect infants and young children from families affected by serious socioeconomic challenges (91).

The overall impact of an effective RSV vaccine in the developing world remains to be determined. Shi et al. estimated that a vaccine against RSV with 80% efficacy would prevent 22,000 in-hospital deaths every year (88). But studies of RSV mortality show sequential seasonal peaks of RSV and pneumococcal disease suggesting a potential synergic association between both pathogens (92), and a third of community deaths during the winter associated with RSV in the only study with information about viral etiologies so far (91). In fact, the recently observed protection conferred by ResVax® against all-cause LRTI and against all-cause LRTI with significant hypoxemia suggest our estimations may require revision in the near future. In fact, detecting RSV using RT-PCR is challenging in underserved settings where deaths typically occur, and testing for it in critically ill patients is infrequent given the absence of specific therapies (93). Moreover, randomized clinical trials and formal studies enhance surveillance and standardize care, perhaps providing an overly optimistic perspective on this problem.

## Breast Milk and RSV Vaccines

Breastfeeding is a critical asset to protect infants against respiratory viruses in developing countries (94). But the potential benefit conferred by antibodies in human milk elicited by maternal immunization is uncertain.

Numerous molecules in breast milk, passively transferred to the baby during lactation, have been postulated to confer protection. Among them, sIgA has been the most widely accepted example of this passive mechanism (95). However, this hypothesis has potential inconsistencies that require further consideration. First, protecting the lung with passively acquired defenses would require frequent “mini” gastric aspirations to coat the nasopharynx, as the predicted persistence of molecules in the upper respiratory tract is short lived (96). Such a mechanism would be highly inefficient, notwithstanding the repetitive risk for a more severe aspiration. Second, babies are often exposed to RSV simultaneously with their mothers (97). This timing is problematic, as Ig protection of the respiratory tract requires high levels of local antibody. Therefore, the situation would demand instantaneous boosting of maternal immunity to timely prevent infection in infants. Because it is not entirely logical to postulate that mothers carry protection in adequate concentrations against all pathogens at all times. Third, studies on RSV and nasal IgA neither exhibited a strong correlation between baseline IgA levels and ARI (98), nor were able to correlate neutralizing activity and anti-RSV IgA levels (99).

But there is another argument that supports the hypothesis that the main mechanism of protection against RSV in human milk is not through passive transfer of sIgA. At least five studies in different populations from different regions and risk condition, described sex differences in breast milk-mediated prevention of severe viral LRTI in children. In all these studies, milk protects female infants better than males (100–104). Therefore, human milk appears to activate a process in the baby. And this process, in line with evolution and preservation of the species, is better suited for females (100–104). It will be interesting to learn whether such benefits can be boosted through RSV immunization.

## Benefits for Pregnant Women from Maternal Immunization

Influenza virus and *B. pertussis* maternal vaccines prevent severe disease in infants, but differ on their ability to affect maternal illness. Influenza virus is known to cause severe disease in pregnant women and its effects extend to influence both timing of delivery and fetal growth (105). Conversely, pertussis in adults is relatively mild (106). Post-vaccine licensure studies will define whether RSV vaccines belong to the first or the second group.

Defining RSV disease burden during pregnancy is challenging. Proper ascertainment of the problem would require very large prospective studies, intense outpatient follow-up, and consequently considerable funding. Therefore, most information today about RSV in pregnant women derives from *post-hoc* analyses of trials evaluating maternal immunogens against flu (107). This strategy is not perfect, particularly due to two important limitations: unlike in flu, fever is not a frequent clinical sign associated with RSV infection in pregnant women (108); and often RSV and influenza seasons do not overlap exactly (109). Then, studies using criteria tailored for influenza-like illness in flu studies to prompt sick visits or collect samples from mothers to study RSV probably underestimates the burden of illness (110). In absence of RSV-targeted studies, we cannot convincingly state today that RSV elicits milder disease than influenza during pregnancy. While RSV in pregnancy elicits mild illness, it can occasionally cause acute respiratory failure (111).

## Strategy-Specific Challenges

Very specific logistical challenges may impact each immunization strategy. Maternal immunization may be most effective with different windows for immunization in pregnant women, should preparations differ. These decisions should be influenced by the levels of pre-existent antibodies in women and will also depend on the quality of prenatal care to maximize immunization rates. In the ResVax® RCT, immunization >30 days before delivery enhanced infant protection. In addition, acceptance of maternal immunization may differ in different populations due to cultural idiosyncrasies. Passive prophylaxis using mAb of extended half-life will require precise characterization of the local RSV season in different regions. This may prove challenging in tropical climates, where seasonality can oscillate by weeks in different years (112). And, importantly, cost of the drug in developing countries may become a critical factor for universal administration. These interventions are most needed in LMIC, where most morbidity and mortality occur. Finally, strategies like intranasal live attenuated RSV vaccine administration may demand a fine balance between optimal immunogenicity and no pathogenicity, require temperature stability for tropical climates, and inaugurate inoculation of RSV vaccines in older infants through a novel route of immunization, that for flu intranasal vaccines is not entirely comfortable.

In summary, the landscape of bronchiolitis and viral pneumonia may be altered directly and indirectly by the interventions under evaluation today. A long list of candidate vaccines (i.e., FIRSV, PFP-2) and mAb (i.e.: motavizumab®, suptavumab®) have failed over the years for different reason to control this feisty virus in young children. But both palivizumab® and the results from ResVax® in LMIC demonstrate that protection against RSV is possible. Just like many physicians in the industrialized world have rarely ever seen a case of measles, pediatricians in 2030 may be entirely unfamiliar with the packed emergency rooms, lack of available beds, or emergency situations that we experience every year due to RSV. Human metapneumovirus, rhinoviruses and human parainfluenza viruses may concentrate a lot more attention. A brighter future may be near.

## Author Contributions

SSA and FPP reviewed the literature, conceived and wrote the manuscript.

### Conflict of Interest Statement

FPP received consulting funds from Novavax, GSK, Pfizer, Sanofi, MedImmune, Janssen, Merck, VirBio, Daiichi Sankyo, and Bavarian Nordic. The remaining author declares that the research was conducted in the absence of any commercial or financial relationships that could be construed as a potential conflict of interest.
